# Targeting Oncogenic Protein-Protein Interactions by Diversity Oriented Synthesis and Combinatorial Chemistry Approaches

**DOI:** 10.3390/molecules16064408

**Published:** 2011-05-27

**Authors:** Andreas G. Tzakos, Demosthenes Fokas, Charlie Johannes, Vassilios Moussis, Eleftheria Hatzimichael, Evangelos Briasoulis

**Affiliations:** 1 Human Cancer Biobank Center, University of Ioannina, Ioannina, GR-45110, Greece; 2 Department of Chemistry, University of Ioannina, Ioannina, GR-45110, Greece; 3 Department of Materials Science and Engineering, University of Ioannina, Ioannina, GR-45110, Greece; 4 Institute for Chemistry and Engineering Sciences, 11 Biopolis Way, Helios, #03-08, Singapore 138667, Singapore; 5 Department of Clinical Hematology, University Hospital of Ioannina, Ioannina, GR-45110, Greece; 6 Medical School, Oncology Section, University of Ioannina, Ioannina, GR-45110, Greece

**Keywords:** diversity-oriented synthesis, combinatorial chemistry, protein-protein interactions, cancer

## Abstract

We are currently witnessing a decline in the development of efficient new anticancer drugs, despite the salient efforts made on all fronts of cancer drug discovery. This trend presumably relates to the substantial heterogeneity and the inherent biological complexity of cancer, which hinder drug development success. Protein-protein interactions (PPIs) are key players in numerous cellular processes and aberrant interruption of this complex network provides a basis for various disease states, including cancer. Thus, it is now believed that cancer drug discovery, in addition to the design of single-targeted bioactive compounds, should also incorporate diversity-oriented synthesis (DOS) and other combinatorial strategies in order to exploit the ability of multi-functional scaffolds to modulate multiple protein-protein interactions (biological hubs). Throughout the review, we highlight the chemistry driven approaches to access diversity space for the discovery of small molecules that disrupt oncogenic PPIs, namely the p53-Mdm2, Bcl-2/Bcl-xL-BH3, Myc-Max, and p53-Mdmx/Mdm2 interactions.

## 1. Introduction

Cancer develops through a multistep complex process that involves a series of genetic and epigenetic alterations which in turn, ultimately, lead to malignant phenotypes [1,2,3,4]. Deciphering of the molecular mechanisms of cancer stirred high expectations for the development of smart drugs that could efficiently inhibit aberrantly functioning cancer-driving oncoproteins [5]. Indeed, targeted drugs substantially improved the therapy of chronic myeloid leukemia, acute promyelocytic leukemia and gastrointestinal stromal tumors, in which cases tumor specific driver oncoproteins were identified and successfully targeted [6,7,8]. However, these tumors make up a minor fraction of worldwide cancer. In the settings of major killing cancers, such as lung, gastric, pancreatic, colon, breast and prostate cancer, the success has been disappointingly limited, not affecting survival rates [9]. Examples of effective targeted therapies that have entered clinical use are: proteasome inhibitor bortezomib which has improved the median survival of multiple myeloma patients [10,11], several tyrosine kinase inhibitors effective against renal and lung cancer [12], mTOR inhibitors [13], and lately, DNA methylation and chromatin modifying epigenetic drugs [14,15]. All this new therapeutic armamentarium has had, unfortunately, little effect on the ultimate clinical outcome of most cancers [16].

The limited efficacy of many first generation targeted drugs, designed to attack specific molecular alterations, is now understood to be a consequence of the complexity of aberrantly functioning of biomolecules in cancer [17,18]. It is therefore imperative to devise and develop novel drug discovery strategies in order to have chances to address the hard kernel of cancer complexity and improve cancer therapy [19,20,21]. Recently, advances towards the understanding of cancer system biology led to consider cancer-related protein-protein interaction networks as appropriate therapeutic targets, although the druggability of this approach is questioned [22,23,24]. Nevertheless, positive results of early studies provided encouraging evidence of selective and efficient interruption of aberrant protein-protein interactions in cancer, opening up a new avenue in cancer drug development [25,26,27].

**Figure 1:**
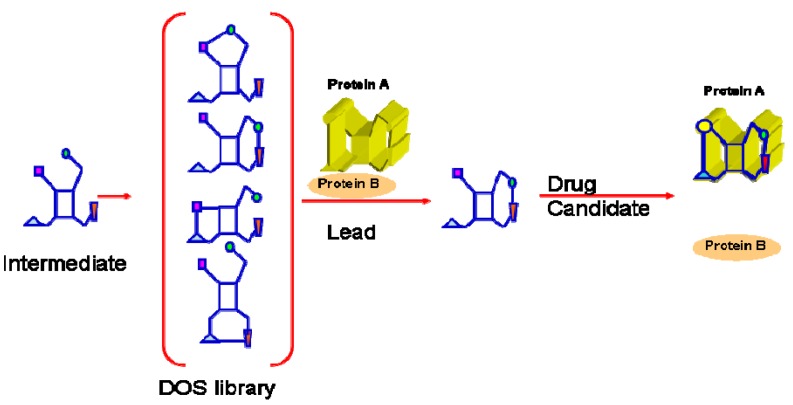
In DOS, libraries of structurally diverse compounds are derived from common intermediates (reaction of the same starting material with different reagents or reaction of different starting materials with common reagents). Different chemical groups are presented with different colors. The binding cavity that is targeted by DOS compounds in a protein-protein interaction is colored in yellow. Optimized DOS compounds can dissociate such PPIs.

Combinatorial Chemistry and Diversity Oriented Synthesis (DOS) [28,29,30,31] are chemical technologies that have been used to generate screening collections which contain various aspects of structural diversity. The main intent of both approaches is to exploit state of the art synthesis and technological advancements. DOS is an evolution of combinatorial synthesis which leverages a forward synthetic planning strategy in order to obtain the most diverse set of molecules in an efficient manner (Figure 1). As an approach, DOS is uniquely different from traditional target oriented synthesis (TOS). TOS requires retrosynthetic planning strategies to manage the sometimes monumental synthetic challenges as well as prioritize the numerous synthetic options en route to a single and usually complex target molecule.

The hypothesis at the conception of DOS was that large collections of molecules derived from combinatorial chemistry were too similar to each other and not novel, diverse or complex enough to be probes for challenging difficult biological targets such as protein-protein interactions (PPIs). DOS evolved to encompass the rapid synthesis of complex small molecules as a compromise between total synthesis and combinatorial chemistry techniques. In the design of DOS libraries, relevant parameters that define compound properties are also taken into consideration, such as the Lipinski rules [32]. Certain deviations from these traditional parameters such as the incorporation of higher molecular weight (MW) compounds are included. The resulting compounds derived from DOS strategies have helped to launch an area of research known as chemical genomics [33]. Several other approaches similar to DOS such as Biology Oriented Synthesis (BiOS) [34,35], Functional Oriented Synthesis (FOS) [36], and Diverted Total Synthesis (DVT) [37] have also emerged with the intent to capture and leverage “natural product like (NPL) features”. There are no generally accepted parameters for what NPL is. However, notable attempts to compare NP collections with other synthetic collections have been described [38,39], providing empirical evidence of higher polarity, decreased hydrophobicity, higher molecular weights, increased stereochemical features and therefore higher sp^3^ content [40], unique molecular architectures and fewer aromatic rings [31]. These strategies are a clever way to address the shortcomings of using unguided combinatorial chemistry techniques [41,42] in the absence of other traditional structure-guided approaches.

**Figure 2:**
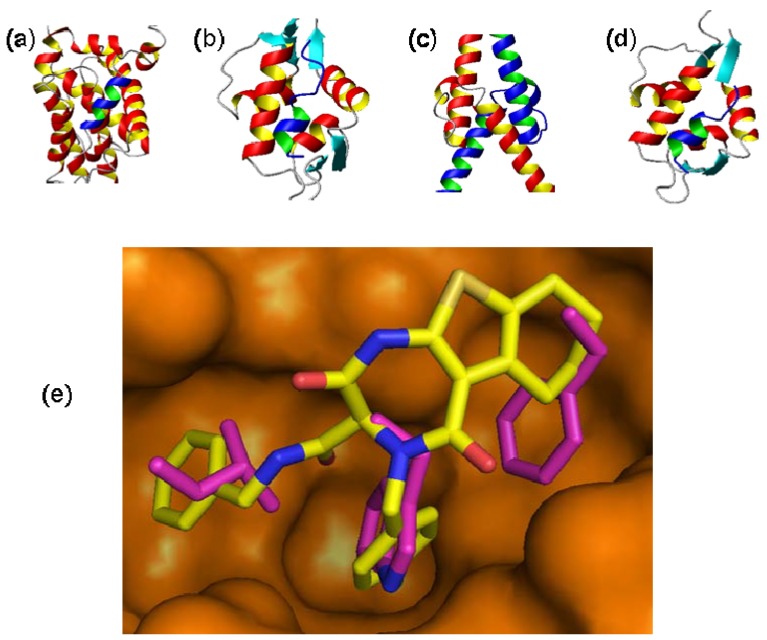
Several three dimensional structures of protein-protein interactions that have been targeted by DOS have been solved by X-ray crystallography or Nuclear Magnetic Resonance spectroscopy (NMR): (**a**) Structure of a helical peptide from the death-promoting region of the Bcl-2-related protein Bak (colored in blue/green) bound to the survival protein Bcl-xL (colored in red/yellow) (pdbid:1BXL); (**b**) Structure of a p53 helical peptide (colored in blue/green) bound to Mdm2 (colored in red/yellow) (pdbid: 1YCR); (**c**) Structure of the basic/helix-loop-helix/leucine zipper (bHLHZ) domains of Myc (colored in red/yellow) and Max (colored in blue/green) heterodimers (pdbid: 1NKP); (**d**) Structure of a 15-residue transactivation domain peptide of human p53 (colored in blue/green) bound to the N-terminal domain of human Mdmx (colored in red/yellow) (pdbid: 3DAB). (**e**) Docking of an inhibitor of p53-Mdm2 interaction, developed from a small focused compound library of 1,4-thienodiazepine-2,5-diones, into the p53-binding site of Mdm2 (pdbid: 1YCR). The inhibitor (colored in yellow) is able to mimic hot spot residues (colored in purple) implicated in the p53-Mdm2 interface [47].

There are a few salient structural characteristics regarding various diversity aspects of DOS that have been previously reviewed [43,44], such as appendage, substitutional, stereochemical, or scaffold diversity. For the most part, each descriptor contributes to the overall shape of the library. Recently, principle moments of inertia (PMI) plots [45] have been used to generally differentiate the shapes of molecules, and progress has been made in evaluating multiple conformations of the same molecule. Frustratingly, little is still known on how to predict or measure the diversity of stereoisomers despite the general acceptance that stereochemistry is a differentiating feature of many drugs [40]. Often, novelty or general characteristics such as NP likeness and/or complexity of the final compounds are considered. In order to steer the finite resources available to synthesize screening collections, there is usually a guiding principle or inspiration used as a nucleus for the ideas. Some more popular strategies are guided by the specific shape of a target enzyme or protein, a privileged structural motif [46], or a chemical methodology that enables access to novel chemical structures. In the following sections, molecules that target oncogenic protein-protein interactions and have been derived directly from DOS libraries or through the use of combinatorial techniques, in confluence with other technologies, are described (Figure 2).

## 2. Targeting Oncogenic Protein-Protein Interactions with Small Molecules

Traditionally, drug development has focused on a small number of protein classes, (*i.e.*, enzymes and receptors), not exceeding more than 1% of the roughly 30,000 unique protein sequences that comprise the human proteome [48]. Protein-protein interactions (PPIs) are key players in numerous cellular processes and it has been estimated that a very high number of them (40,000 to 200,000) exist in the human interactome. Since aberrant interruption of this complex network provides a basis for various disease states, a fine-tuning of these binding events *via* small molecule interactions has emerged as a rather important strategy for human therapeutics. Given the biological complexity of PPIs, the discovery and optimization of small molecules provides a significant challenge for drug development. A recent analysis of the network characteristics and interface properties of cancer-related proteins revealed that these are distinct from non-cancer proteins [49,50]. Specifically, it was shown that cancer-related proteins tend to interact with their partners through distinct interfaces, corresponding mostly to multi-interface hubs [49]. In addition, it was shown that they possess more planar, more hydrophilic, but smaller binding sites compared to non-cancer proteins, indicating low affinity and high specificity of the cancer-related interactions [49]. Such decoding is of importance only to reveal the details of specific binding regions for cancer-related protein interactions and may be utilized to formulate the drug development process accordingly. An *in vivo* proof of principle on the efficacy of protein-protein interaction inhibitors as anticancer drugs exists [26,51,52].

Although the importance of PPIs in drug development is well documented, PPIs have been extremely challenging targets. However, it should be noted that traditional approaches, such as high-throughput screening, have been successfully exploited in developing potent selective PPI antagonists. For instance, the discovery of Nutlins, the *cis*-imidazoline analogs that target the MDM2-p53 protein-protein interaction on the intent to reactivate p53, as well as the discovery of potent small molecules inhibitors that interfere with bcl-2 protein-protein antiapoptotic interactions, constitute such examples [51,53,54,55,56,57,58]. Navitoclax, a targeted high affinity inhibitor of Bcl-2 has already been evaluated in phase I and Nutlin-3 is currently about to enter early clinical evaluation [59,60].

Numerous factors have hindered a fruitful exploitation of PPIs as potential intervention points for the development of anticancer agents. For instance, PPI surfaces are large (750 Å–1,500 Å) [61] and devoid of deep interventions [22]. Affinity is achieved from the accumulation of numerous weak interactions. Therefore, it is inherently difficult for a small molecule to compete for binding on such an extensive interface composed of a large number of individual and complimentary interactions. To complicate the situation further, the inherent malleability of proteins to accommodate surface complementarity significantly handicaps structure-guided approaches. Furthermore, the small number of available assays to discriminate real from artifactual binding could hinder the development of small molecule antagonists for PPIs. However, despite the aforementioned difficulties, important progress has nonetheless been achieved towards the discovery of PPI antagonists. Indeed, this became evident upon analysis of protein-protein interfaces which showed that a centralized region of residues, the so called “hot-spots” [62], mediate all the key interactions that contribute to the binding affinity and presents comparable dimensions to the size of the small organic molecule. Such observations have recently challenged the traditional thought that PPIs are “undruggable” targets and numerous small-molecule inhibitors of PPIs are now in clinical trials [48,61]. Additionally, several strategies have surfaced regarding the discovery of small-molecule modulators of PPIs (for an insightful review see ref. [63]). Thus, disruption of oncogenic PPIs with small molecules might lead to a new class of anticancer therapeutics. Since *α*-helix-mediated PPIs are involved in a wide array of cellular signaling pathways, discovery of cell permeable and bioavailable small molecule inhibitors of these interactions could pave the way in the field. In many PPIs, short helical peptides play an important role as a recognition motif, where side chains at *i*, *i*+3 or *i*+4, and *i*+7 positions often become a critical determinant for PPIs [64,65]. Indeed, DOS has been successful in the discovery of lead compounds targeting *α*-helix-mediated PPIs in numerous cases such as: the complex between the Bcl-2-related proapoptotic protein Bak bound to the survival protein Bcl-xL (Figure 2a), the p53 derived helical peptide bound to murine double minute 2 (Mdm2) (Figure 2b), the complex of the basic/helix-loop-helix/leucine zipper (bHLHZ) domains of Myc and Max heterodimers (Figure 2c), and the complex of a 15-residue transactivation domain peptide of human p53 bound to the N-terminal domain of human Mdmx (Figure 2d). These applications will be analyzed in the following sections.

## 3. Antagonists of p53-Mdm2 Interactions

The tumor suppressor p53 is a well recognized target in cancer drug discovery which could offer new therapeutic opportunities [66]. The activation of wild-type p53 in human tumors with small molecules that antagonize Mdm2 appears to be a promising strategy in the treatment of cancer [67]. The disruption of the p53-Mdm2 interaction is usually accomplished by peptides, foldamers and peptoids (*α*-helical transactivation domain), chemical entities aiming mainly to mimic the p53 fragment and the Mdm2-binding site [68]. Also, the design of small molecules with appropriate physicochemical properties (*i.e.*, bioavailability, aqueous solubility, stability) could enable the discovery of efficient Mdm2 antagonists [69].

### 3.1. Antagonists of p53-Mdm2 Interactions Based on 1,4-thienodiazepine-2,5-dione Based Core Structures

**Figure 3:**
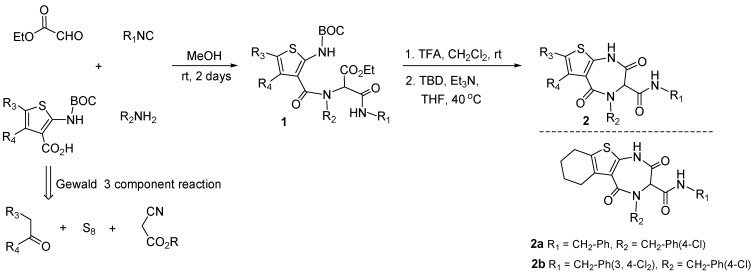
Application of an Ugi-4CR in the discovery of p53-Mdm2 antagonists.

A peptidomimetic strategy was employed to synthesize small molecule p53-Mdm2 antagonists taking advantage of an Ugi-deprotection-cyclization sequence. The Ugi reaction has been exploited in combinatorial chemistry because it combines four separate components to make one scaffold. This provides easy access to appendage diversity around one single scaffold. In this case, the scaffold is a peptidomimetic 1,4-thienodiazepine-2,5-dione and was envisioned to act as an *α*-helix mimetic and to disturb the p53-Mdm2 interaction. A small library of 18 diverse thienodiazepine-2,5-diones with general structure **2**, selected from a large virtual library, was prepared in one pot by solution phase synthesis via an Ugi-deprotection-cyclization strategy [47]. Condensation of 2-aminothiophene carboxylic acids, ethyl glyoxalate, amines and isonitriles, gave the Ugi-4CR product **1**. Boc deprotection followed by TBD (1,5,7-triazabicyclo [4,4,0]dec-5-ene) mediated cyclization produced azepinediones **2**. Access to a diverse set of 2-aminothiophene carboxylic acids can result from the Gewald three component reactions (Figure 3). 

Library screening, following two complimentary techniques, found 1,4-thienodiazepine-2,5-dione **2a** and **2b** to antagonize the p53-Mdm2 protein interaction. Both compounds were found to antagonize the p53-Mdm2 interaction in a fluorescence polarization assay, exhibiting a dose dependent effect to compete with a p53-like peptide. Diazepinediones **2a** and **2b** inhibited Mdm2 with inhibition constant (K_i_) values of 40 μM and 45 μM, respectively. Also, in a Nuclear Magnetic Resonance (NMR) competition assay performed on the Mdm2⁄p53 complex, compounds **2a** and **2b** were found to dissociate the Mdm2⁄p53 complex with *K*_d_ values of 30 ± 20 μM and 10 ± 6 μM, respectively.

This study demonstrated that the 1,4-thienodiazepine-2,5-dione scaffold is bioisosteric to the well known benzodiazepinediones and perhaps by correlation, may be a bioisosteric privileged structure. Enumeration of the scaffold followed by plotting MW *vs.* TPSA, compared to available benzodiazepine compounds through eMolecules [70], suggests that there is a large potential diversity which can be accessed based on the developed chemistry.

## 4. Targeting Anti-Apoptotic Members of the Bcl-2 Family Proteins

The Bcl-2 (B-cell lymphoma) family proteins regulate the equilibrium between cell proliferation and cell death (apoptosis) through complex protein-protein interactions. This family is composed of antiapoptotic and proapoptotic members. The antiapoptotic members contain four Bcl homology (BH) domains (BH1−BH4) and include Bcl-xL, Bcl-w, Bcl-2, Bcl2-A1 and Mcl-1, whereas the proapoptotic members contain either a single BH3 domain (BH3-only) (Puma, Bad, Bik, Bid, Bim) or three (BH) domains (BH1−BH3) (Bak, Bax). Apoptosis, or programmed cell death, is a highly controlled biological mechanism regulating the removal of aged, damaged, and unnecessary cells [71,72,73,74,75]. Aberrations in this equilibrium circuit can allow transformed cells to evade death and become resistant to cytotoxic therapies. Hence, the Bcl-2 pathway has been a compelling target for drug development for more than two decades. The critical event in Bcl-2 family signal propagation is the direct association of a protein containing a BH3 death domain with a multi-domain Bcl-2 family member. The antiapoptotic proteins bind their proapoptotic counterparts and sequester them from the cellular environment, thus inhibiting the apoptosis process. The up regulation of antiapoptotic members of this family (Bcl-2, Bcl-xL) is observed in many cancers. This overexpression prevents the activation of apoptosis and can protect cancer cells, favoring their proliferation and survival when exposed to anticancer compounds [76,77,78]. Therefore, the design of small molecules that bind the BH3 domain of antiapoptotic proteins and inhibit PPIs, can offer new strategies in cancer therapy [79]. Analysis of the three-dimensional structures of antiapoptotic Bcl-2 family proteins showed how these specific proteins interact with their proapoptotic counterparts [76,77,78]. It was revealed that the binding cavity for the proapoptotic molecules was an elongated hydrophobic crevice of approximately 20 Å, called BH3 binding groove. The understanding of these protein-protein interactions has opened new directions for rational design of novel inhibitors.

### 4.1. Discovery of Novel Bcl-2 Inhibitors Based on Rigid Pyridone Scaffolds

**Figure 4:**
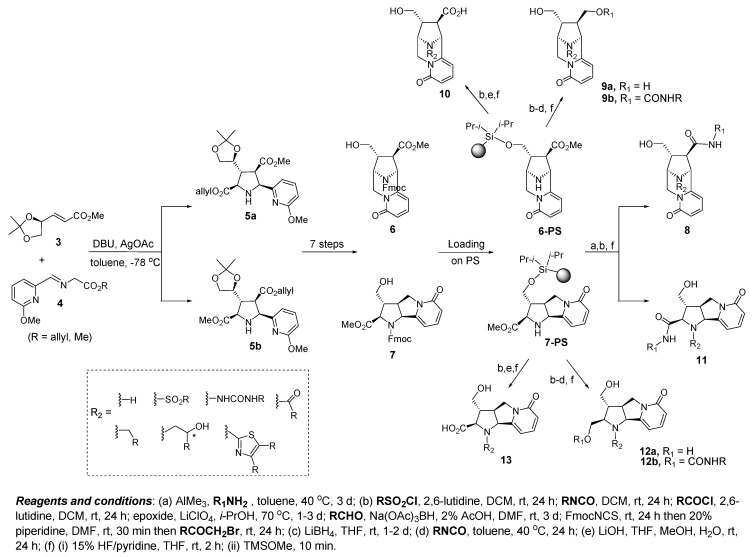
Discovery of Bcl-2 inhibitors based on DOS of pyridone core structures.

Screening of a DOS library, containing 15,000 compounds inspired by the tricyclic alkaloid natural product cytisine containing the privileged structural pyridone motif, led to the identification of novel inhibitors of Bcl-2 [80]. The stereochemical and skeletal diversity is accomplished by taking advantage of highly substituted pyrrolidines **5a** and **5b**, accessed from a stereoselective [3+2] dipolar cycloaddition that then diverges into two distinct and novel tricyclic scaffolds **6** and **7** (Figure 4).

Appendage diversity was exploited by loading the scaffolds **6** and **7** onto a solid phase support to provide **6-PS** and **7-PS** and then employing a split and pool strategy. In this way, a sparse matrix could be utilized by exploring all combinations of capping strategies including changing the oxidation state of the handles. For example, amidation of the methyl ester of **6-PS** under Weinreb conditions followed by capping of the secondary pyrrolidine nitrogen with a series of electrophiles, gave access to a diverse set of pyridones with general structure **8** after fluoride mediated resin cleavage. Alternatively, capping of the secondary pyrrolidine nitrogen first followed by ester reduction, resulted in alcohol **9a** after resin cleavage. Also, treatment of the intermediate resin-bound primary hydroxyl with a series of isocyanates, produced carbamates **9b** after release from the resin. Furthermore, a number of compounds with general structure **10** were prepared from **6-PS** after derivatization of the pyrrolidine nitrogen and ester hydrolysis. Pyridone **7-PS** gave access to chemotypes **11**, **12a**,**b** and **13** as well, by application of the same protocols.

**Figure 5:**
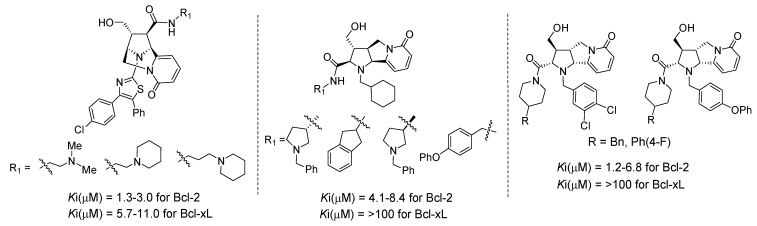
Discovery of selective inhibitors of Bcl-2 based on tricyclic pyridone scaffolds.

The library compounds were screened for binding affinity against Bcl-2 and Bcl-xL in traditional solution based competition binding assays, formatted for HTS analysis, against a fluorescently tagged BH3 peptide. The hit rate from this library screen was 1.1% and 0.2% against Bcl-2 and Bcl-xL respectively, with the best inhibitors having single digit micromolar activities [80]. The most potent compounds identified in the bridged bicyclic pyridone chemotype **8** were those containing a diamine at R_1_ and a chloro-substituted diphenyl 2-aminothiazole at R_2_ (Figure 5). Both enantiomers in this series were equally active against Bcl-2, perhaps indicative of non-specific binding. They also displayed inhibitory activity against Bcl-xL except for compounds which lacked the diamine at R_1_. The most active compounds derived from the tricyclic pyridone chemotype **11** were the products of reductive alkylation and Weinreb amidation. For one enantiomeric series there was a distinct preference at R_2_ for cyclohexyl carboxaldehyde in combination with primary amines containing a hydrophobic aromatic ring at R_1_ (Figure 5). For the enantiomeric series (**ent-11**), 3,4-dichlorobenzaldehyde as well as 4-phenoxybenzaldehyde were preferred at R_2_ in combination with benzyl or phenyl substituted piperidines at R_1_ (Figure 5). This difference in activity for the two enantiomeric series suggests more specific binding as compared to the 2-aminothiazole compounds. In both cases the active compounds in this series were selective for Bcl-2 over Bcl-xL. Notably, this example highlights that a purely chemistry driven approach to novel alkaloid scaffolds can lead to the discovery of inhibitors of protein-protein interactions. Furthermore, this chemistry approach provides the flexibility to design specific appendage diversity enabling a quick understanding of the SAR. 

### 4.2. Discovery of Bcl-2 Inhibitors from an Isoxazolidine Library

**Figure 6:**
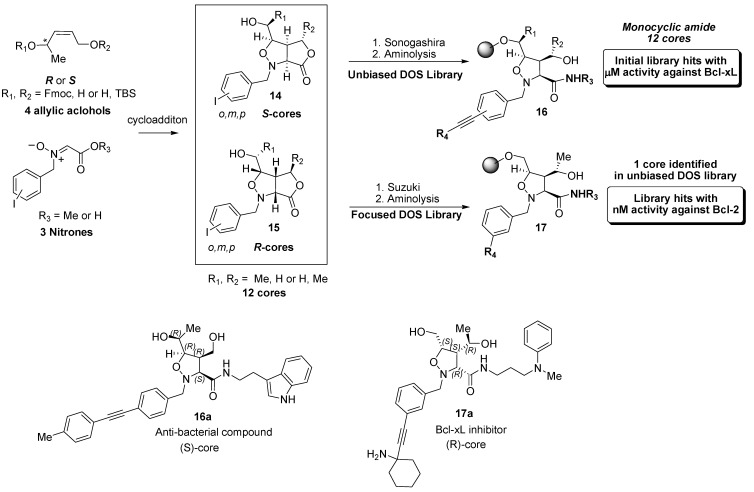
Isoxazolidine based Bcl-2 inhibitors.

An unbiased DOS library which incorporated appendage, scaffold, and stereochemical diversity has previously been reviewed [81] (Figure 6). Notably, the library was screened in multiple assays and in different therapeutic areas which resulted in the discovery of low micromolar hits in both Bcl-xL **17a** as well as an antibacterial hit **16a**. The stereochemical importance is underscored by the fact that the respective hits were derived from the enantiomers of related cores. In order to access the structural and stereochemical diversity of this library, a diastereoselective 1,3-dipolar cycloaddition/acyclation reaction between each enantiomer of the allylic alcohols and various nitrone carboxylic acids was employed to generate 12 scaffolds represented by the general bicyclic structures **14** and **15**. A small library of bicyclic compounds was synthesized and a larger monocyclic isoxazolidine library **16** was obtained from Sonogashira coupling of the aromatic iodides and substituted terminal alkynes, followed by aminolysis of the lactone. Notably, the lead compound was identified from only this subset of the entire library. Further structural diversity was obtained *via* cleavage of the N-O bond to provide a library of α-amino amide compounds (not shown). Despite the similarity of the appendage diversity, this subtle change resulted in no Bcl-2 activity.

Interestingly, a recent patent application has been published [82] which indicates that hit to lead activities have identified biaryl analogs **17** where potency has been pushed to nanomolar levels and optimized for Bcl-2 instead of Bcl-xL inhibition. These biaryl compounds were not part of the initial screening library and exemplify how the library design can be exploited quickly to optimize hit to lead efforts by simply replacing the sonogashira couling with the Suzuki reaction.

### 4.3. Discovery of Bcl-xL Antagonists Resulting from Oxabicyclic Scaffolds

**Figure 7:**
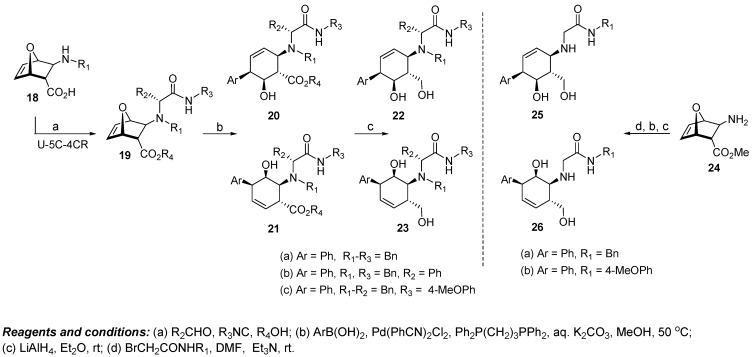
Design of Bcl-xL antagonists through structural modification of oxabicyclic scaffolds.

A strategy employing iterative molecular docking of unique oxabicyclic scaffolds, combined with NMR studies, was employed in an attempt to identify antagonists of the antiapoptotic protein Bcl-xL, a member of the Bcl-2 family of survival proteins [83]. The pluripotent scaffold **19**, derived from chiral *β*-amino acid **18** via an Ugi-five center-four component reaction (U-5C-4CR), was selected for this study because of its ability to provide easy access to new cyclohexene cores where the olefin and the substitution pattern of the ring can be varied. For this example, although the actual scaffold (cyclohexene ring) was not changed, the shape of the core is varied by the regio and appendage diversity to provide enough differentiation in structure. A set of virtual libraries were designed based on these scaffolds and then tested *in silico* for their capacity to bind to the BH3 binding groove of Bcl-xL. Analysis of docking calculations and comparison of all tested compounds revealed that scaffolds **20** and **21** can be used in the design of promising molecules, particularly the alcohol series **22** and **23**. Therefore, in this particular case, the potential diversity of the oxabicyclic scaffold as it relates to the BH3 binding groove was narrowed on the basis of computational results, and compounds **22a**,**c** and **23a-c** as well as their enantiomers (**ent-22a**,**c** and **ent-23a-c**) were identified as potential antagonists (Figure 7). Cyclohexenols **20** and **21** were prepared as a regioisomeric mixture from the palladium catalyzed ring opening of the chiral oxabicyclic scaffold **19** in the presence of aryl/heteroaryl boronic acids. Ester reduction resulted in diols **22** and **23**, respectively. 

When compounds **22a**,**c**, **23a-c**, **ent-22a**,**c**, and **ent-23a-c** were tested for their ability to bind Bcl-xL in NMR-based binding assays, the results were inconclusive due to the low solubility of the compounds in the solvent used for the NMR experiments. Therefore, the more hydrophilic analogs **25** and **26** were chosen for the NMR based screening. Computational screening identified compounds **25a**,**b**, **26a**,**b** as well as their enantiomers **ent-25a**,**b** and **ent-26a**,**b** to possess the structural elements required for binding. Access to chemotypes **25** and **26** was obtained through conversion of amino ester **24** via an alkylation reaction sequence with bromoacetamides. Experimental NMR data showed weak binding of this group of compounds with Bcl-xL. Unfortunately, the corresponding fluorescence polarization assay, using a fluoresceinated Bak peptide with the full length protein, did not show any appreciable displacement at 200 μM concentration. Therefore, despite the unique access to the oxabicyclic scaffold via the Ugi reaction, promising computational studies and NMR work, the oxabicyclic scaffold did not provide the intended results. An intriguing experiment would be to exploit the developed methodology to provide a more diverse set of compounds to see if a better hit would be derived. Moreover, the resulting compounds should be tested in other biochemical assays to see if the oxabicyclic scaffold is more suited for a different target.

## 5. Isoindoline Based Antagonists of the Myc-Max Protein-Protein Interaction

**Figure 8:**
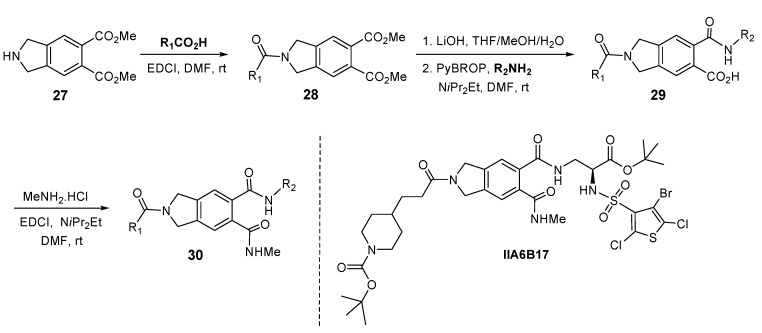
Isoindoline-based antagonists of the Myc-Max protein-protein interaction.

Disruption of the oncogenic PPIs between the Myc and Max transcription factors by small molecules, should enable the discovery of valuable probes for dissecting the roles of these transcription factors in cancer and for evaluating their potential as new therapeutic targets. Myc is aberrantly activated in a number of human cancers [84,85,86,87,88,89,90] and acts by heterodimerization with Max via their helix-loop-helix leucine zipper domains, a process that leads to the transcription of Myc target genes. In an effort to identify small molecules that antagonize the Myc-Max heterodimerization process, a screening collection of approximately 7,000 small organic molecules were used in a FRET assay. A subset of the 7,000 compounds contained a 240 membered privileged structure library based on an isoindoline scaffold: four isoindoline compounds were identified as hits. One hit originating from the diamide-acid chemotype **29** and three compounds from the triamide **30**, were identified as PPI inhibitors between the Myc and Max transcription factors [91]. The library design is mainly focused on introduction of appendage diversity through sequential elaboration of the trifunctional isoindoline scaffold **27** by solution phase parallel synthesis. The objective was to identify nonpeptide RGD-based antagonists [92]. The appendage diversity was limited to amine and acid building blocks to make amides. Amidation of **27** with a set of carboxylic acids gave access to amide **28**. Ester hydrolysis to diacid, followed by amidation with a set of amines, resulted in 120 diverse diamides **29**. Sequential amidation with MeNH_2_.HCl produced 120 triamides of general structure **30** (Figure 8).

The initial hits were found to be active in ELISA and electrophoretic mobility shift assays (EMSA) as well, where isoindoline IIA6B17 stood out as the most active compound (ELISA IC_50_ ≈ 125 μM; EMSA IC_50_ ≈ 50 μM). Also, two of these hits inhibited cell focus formation in Myc-transformed chicken embryo fibroblasts (IIA6B17 IC_50_ = 15–20 μM). Although low micromolar inhibitors were identified in this study, this work highlighted the feasibility of inhibiting oncogenic transcription factor PPIs with small molecules [93,94]. In addition, a few diamide and triamide compounds **29** and **30** have also exhibited cytotoxic activity in a leukemia mouse L-1210 assay [92]. Further elaboration of the appendage diversity could be achieved on this particular isoindoline scaffold by incorporating other functionality besides amides, thus leading to a wider selection of chemotypes for screening in other therapeutic areas.

## 6. Discovery of Dual Mdmx/Mdm2 Inhibitors Based on Pyrrolopyrimidine Scaffolds as *A*-Helix Mimetics

A library of 900 compounds based on a pyrrolopyrimidine scaffold as an *α*-helix mimetic, was prepared by solid phase parallel synthesis in the hope to discover small molecules able to disrupt the interaction between p53 and Mdmx/Mdm2 [95]. Mdmx is overexpressed in many cancers and functions as a major regulator of p53 activity (both independently and synergistically with Mdm2). Thus, the development of Mdmx inhibitors that could act solely on Mdmx or on both Mdmx and Mdm2 is highly desirable but still remains challenging [96,97,98].

**Figure 9:**
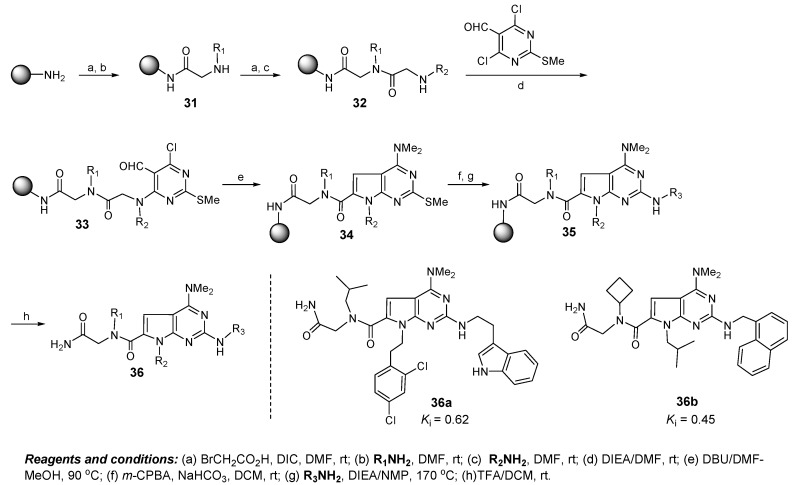
Design of dual Mdmx/Mdm2 inhibitors based on pyrrolopyrimidine scaffolds.

The pyrrolopyrimidine based diversity library was inspired by a natural *α*-helical motif and was designed to explore the appendage diversity of specific vectors around the scaffold. The library synthesis commenced with the efficient preparation on Rink resin of the dimeric peptoid **32** to incorporate diversity elements R_1_ and R_2_ through an iterative bromacetylation followed by displacement of the bromide with primary amines (Figure 9).

Coupling of **32** with 4,6-dichloro-2-(methylthio)-pyrimidine-5-carbaldehyde gave pyrimidine **33** which was cyclized to pyrrolopyrimidine **34** with a concomitant dimethylamination. Oxidation of the thiomethyl ether group to sulfone, followed by substitution with a wide range of amines, gave a diverse set of pyrrolopyrimidines **36** after cleavage from the resin. The library molecules were screened at ~40 μM concentration for their ability to displace a Rhodamine-labeled 15-mer p53 peptide from Mdmx protein by a fluorescence polarization assay. Compounds **36a** and **36b** were the two most active agents identified in the screen and effectively inhibited the binding of p53-Mdmx with *K*_i_ = 0.62 and 0.45 μM respectively, comparable with that of a 15-mer p53 peptide (*K*_i_ = 0.8 μM). They were also found to inhibit the p53-Mdm2 interaction with *K*_i_ = 0.62 and 0.84 μM respectively, similar to the binding affinities for Mdmx, suggesting that pyrrolopyrimidines **36a** and **36b** act as dual inhibitors of Mdmx- and Mdm2-p53 interactions.

This library highlights the importance of a specific privileged scaffold or motif. Furthermore, stereochemistry or scaffold diversity was not a defining diversity aspect. Using a specifically designed and very rigid scaffold enabled the exploration of the appendage diversity, resulting in the successful discovery of an inhibitor of Mdmx/Mdm2. Potentially, this approach could be utilized to generate similar libraries and could serve as a useful tool in the discovery of inhibitors of other *α*-helix-mediated PPIs.

## 7. Future Directions

Today the combined efforts of the biotech and pharmaceutical industry as well as governments in drug discovery have yielded improved technologies in several domains: automation, stereoselective methodologies in organic synthesis, assays development, analysis of genetic targets, computational strategies, structural biology, *etc*. However, they have been unable to capitalize and integrate these technologies effectively enough to improve the success of drug discovery in targeting oncogenic protein-protein interactions. For instance, various problems can appear in biochemical screening assays that may hamper the correct validation of PPI inhibitors, including aggregator agents [99,100], reactive false positives [101], frequent hitters [102], and warhead-containing agents [103]. Thus, despite the fact that combinatorial chemistry and diversity oriented synthesis have provided a few hits and leads, there is still a need for novel advancements in order to diagnose artifact pitfalls early on [99,100,101].

DOS is training us to think about diversity and to think forward on how we can access unique chemical space. Combining this with combinatorial know how has provided an interesting approach in building screening decks for drug discovery. This is an important objective since traditional approaches alone are falling short on providing small molecules that modulate key protein-protein interactions important for the regulation of cancer. However, the development of such libraries is still costly and time consuming and it would be a notable improvement to direct our collective synthetic resources towards the exploration of chemical space around specific scaffolds which regulate cancer-related proteins. Recent work on network characteristics and interface properties of cancer-related proteins revealed a distinct trend in comparison to non-cancer proteins [49,50]. Novel cheminformatic tools have been put into place to facilitate the analysis of protein-protein interfaces with regard to their suitability for small molecule drug design [104,105]. Nonetheless, knowing specifically what chemical matter is most relevant is still a far reaching objective. Such discriminative capabilities could be used to rationally design focused libraries. Until then, exploring chemical space to find unique and novel starting points may be our best chance to find druggable chemical matter for these important and challenging protein-protein interactions.
